# Closer look at circulating nitric oxide levels and their association with polycystic ovary syndrome: A meta-analytical exploration

**DOI:** 10.18502/ijrm.v22i12.18061

**Published:** 2025-01-31

**Authors:** Seyed Sobhan Bahreiny, Akram Ahangarpour, Elnaz Harooni, Mahdi Amraei, Mojtaba Aghaei, Reza Mohammadpour Fard

**Affiliations:** ^1^Student Research Committee, Ahvaz Jundishapur University of Medical Sciences, Ahvaz, Iran.; ^2^Medical Basic Sciences Research Institute, Physiology Research Center, Department of Physiology, School of Medicine, Ahvaz Jundishapur University of Medical Sciences, Ahvaz, Iran.; ^3^Department of Immunology, School of Medicine, Ahvaz Jundishapur University of Medical Sciences, Ahvaz, Iran.

**Keywords:** Polycystic ovary syndrome, Nitric oxide, Oxidative stress, Meta-analysis.

## Abstract

**Background:**

Polycystic ovary syndrome (PCOS) casts a wide shadow over the reproductive health of millions of women worldwide, emerging as one of the most complex and multifaceted endocrine disorders. In addition, nitric oxide (NO) stands out as a pivotal signaling molecule, orchestrating a symphony of physiological processes.

**Objective:**

This meta-analysis aims to elucidate the association between NO levels and PCOS, investigate the potential of NO as a biomarker for PCOS diagnosis, and evaluate its clinical significance.

**Materials and Methods:**

A systematic review was conducted in several electronic databases, including PubMed, Web of Science, Cochrane Library, Scopus, EMBASE, and Google Scholar, to identify relevant studies published up to January 2024. Standardized mean difference and 95% CI were calculated using a random effects model to assess the overall effect size. Meta-regressions and subgroup analysis were performed to investigate sources of heterogeneity.

**Results:**

A meta-analysis of 14 studies with 1171 participants showed that NO levels were significantly lower in the PCOS group than in the control group. The pooled analysis yielded a standardized mean difference of -0.482; 95% CI: -0.908 to -0.056; p = 0.027. Subgroup analyses further demonstrated variations in NO levels between different PCOS phenotypes and in relation to metabolic parameters.

**Conclusion:**

This meta-analysis provides evidence for an association between PCOS and dysregulated NO levels and suggests a potential role of NO as a biomarker in the diagnosis and pathogenesis of PCOS.

## 1. Introduction

Polycystic ovary syndrome (PCOS) encompasses a spectrum of metabolic and endocrine derangements that impose significant health concerns on women of childbearing age, often serving as a precursor to a variety of life-impacting co-morbidities (1). Situated at the intersection of reproductive biology and metabolic homeostasis, PCOS challenges medical understanding with its multifactorial etiology, diverse phenotypic expressions, and complex pathophysiological pathways underpinned predominantly by hyperandrogenism and ovarian dysfunction (2).

Recent advancements in the understanding of hyperandrogenism have highlighted its intricate etiology, which may involve contributions from fetal development, maternal factors, and placental influences (3, 4).

This nuanced view of its genesis opens gateways to personalized therapeutic approaches that address the patient's unique disease matrix. The ominous constellation of obesity, insulin resistance, hyperglycemia, and associated cardiovascular risk factors further emphasizes the systemic impact of PCOS and its position as more than a reproductive disorder (5, 6).

Central to our investigation is the role of oxidative stress (OS) a pervasive player in the pathogenesis of PCOS. As the body grapples with the discord between the generation of reactive oxygen species (ROS) and its inherent antioxidant defenses, the resultant OS becomes a critical focus for therapeutic intervention (7, 8).

Nitric oxide (NO) emerges as a pivotal mediator within this redox landscape, straddling roles in vascular regulation and signal transduction, thus implicating it in both the symptomatic presentations and long-term sequelae of PCOS (9). Mounting evidence suggests that the dysregulation of NO synthesis pathways, encompassing the 3 isoforms of NO synthase ([NOS] endothelial, inducible, and neuronal), may have paradoxical implications for PCOS pathology (10).

**Figure 1:**
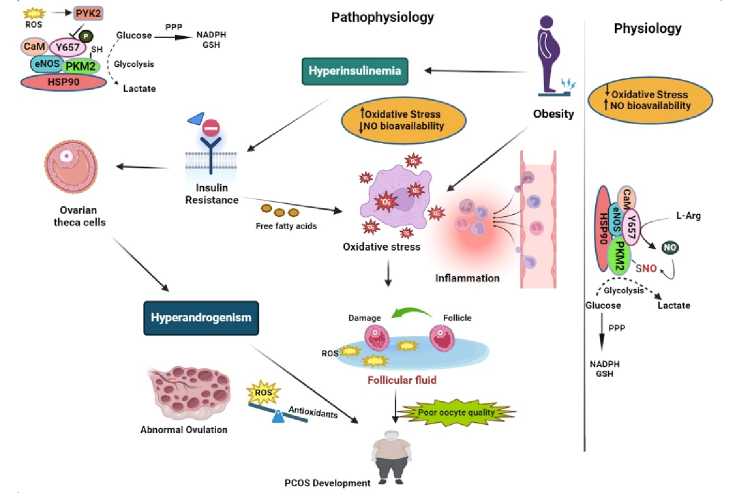
Correlation between NO and PCOS under physiological and pathophysiological conditions.

This dual-faceted role of NO is highlighted by its vascular protective functions courtesy of endothelial NOS (eNOS)-derived NO, juxtaposed against the noxious sequel of inducible NOS (iNOS) over-expression under chronic inflammatory stimuli, commonly witnessed in PCOS phenotypes characterized by insulin resistance (11) (Figure 1). Thus, investigation into serum NO levels in PCOS patients has disclosed a conflicting landscape, with some studies indicating reduced levels implicating vascular dysfunction while others suggest augmented concentrations reflective of chronic inflammation (12, 13). The intricate dance between NO and ROS and their collective influence on the body's redox state presents a potential avenue for diagnostic biomarker development and therapeutic targeting. Given the pivotal role of NO in both the preservation of vascular homeostasis and the exacerbation of oxidative pathology, an opportunity arises to probe deeper into its relationship with PCOS (14–16). To elucidate the enigmatic nature of NO's dual role in PCOS pathophysiology and demystify its prognostic potential as a biomarker, we have embarked on a systematic review and meta-analysis, critically examining observational studies that hinge on this molecule's dynamic within the disorder.

This comprehensive analysis aims to sift through the contradictory evidence and provide a quantitative synthesis of NO levels in women with PCOS. This offers insights into its diagnostic robustness and prognostic value in this prevalent condition. Previous meta-analyses in this area were often based on older studies, with limited subgroup analyses, and lacked rigorous bias assessment and meta-regression techniques. In contrast, our study incorporates a larger, updated dataset. It applies advanced analytical methods including detailed subgroup analyses (age, body mass index [BMI], sample type, and ethnicity), meta-regression, and thorough bias evaluation to deepen our understanding of NO's role in PCOS. By exploring these dimensions, this meta-analysis aims to definitively characterize the interaction between NO and PCOS, paving the way for future personalized treatment strategies that address the underlying oxidative and endothelial disruptions in this complex syndrome.

## 2. Materials and Methods

### Protocol and registration

The systematic review and meta-analysis protocol were registered with “Prospective International Registration of Systematic Reviews" (PROSPERO) CRD42024507048. The systematic reviews were reported according to the 2020 Preferred Reporting Items for Systematic Reviews and Meta-Analyses (PRISMA) statement (17).

### Eligibility criteria

This review only included studies that focused on humans and used observational study designs such as cross-sectional, cohort, or case-control based on population, intervention, comparator, and outcome (PICO) criteria (Table I). The scope of the review was limited to observational studies that examined women aged 18 yr or older who had been diagnosed with PCOS. The diagnostic criteria applied in identifying PCOS patients were the Rotterdam Criteria or National Institutes of Health Criteria. Studies having insufficient data, as well as those that were reviewed, letters to the editor, reports, or were not accessible, were excluded from this review.

### Literature search

Valid medical databases such as PubMed, Web of Science, EMBASE, Scopus, and Cochrane Library (CENTRAL) were searched for the literature review in January 2024. The search terms were defined and searched by a medical librarian and medical professionals, without any time and place restrictions. Search terms were supplemented by searching for available gray studies such as governmental and academic studies. Also, the reference list of all articles related to the snowball method was searched for more comprehensiveness. The keywords “nitric oxide", “polycystic ovary syndrome", and “PCOS" were selected as the primary keywords based on mesh in PubMed. Full details of the search strategy are available in table I. All records reported in the literature search were uploaded to Comprehensive Meta-analysis v3.7z software.

**Table 1:** Search strategy overview (up to January 2024)

**Database**	**Query**
**PubMed**	(“polycystic ovary syndrome"[Title/Abstract] OR (“ovary syndrome"[Title/Abstract] AND “Polycystic"[Title/Abstract]) OR (“Syndrome"[Title/Abstract] AND “polycystic ovary"[Title/Abstract]) OR “stein leventhal syndrome"[Title/Abstract] OR “stein leventhal syndrome"[Title/Abstract] OR (“Syndrome"[Title/Abstract] AND “Stein-Leventhal"[Title/Abstract]) OR “sclerocystic ovarian degeneration"[Title/Abstract] OR (“ovarian degeneration"[Title/Abstract] AND “Sclerocystic"[Title/Abstract]) OR “sclerocystic ovary syndrome"[Title/Abstract] OR “polycystic ovarian syndrome"[Title/Abstract] OR (“ovarian syndrome"[Title/Abstract] AND “Polycystic"[Title/Abstract]) OR “polycystic ovary syndrome 1"[Title/Abstract] OR “sclerocystic ovaries"[Title/Abstract] OR (“Ovary"[Title/Abstract] AND “Sclerocystic"[Title/Abstract]) OR “sclerocystic ovary"[Title/Abstract] OR “pcos"[Title/Abstract] OR “pco"[Title/Abstract]) AND (“nitric oxide"[Title/Abstract] OR (“Nitric"[Title/Abstract] AND “Oxide"[Title/Abstract]) OR (“nitric oxide"[Title/Abstract] AND “Endothelium-Derived"[Title/Abstract]) OR “endothelium derived nitric oxide"[Title/Abstract] OR (“nitric oxide"[Title/Abstract] AND “Endothelium-Derived"[Title/Abstract]) OR “endogenous nitrate vasodilator"[Title/Abstract] OR (“nitrate vasodilator"[Title/Abstract] AND “Endogenous"[Title/Abstract]) OR (“Vasodilator"[Title/Abstract] AND “endogenous nitrate"[Title/Abstract]) OR “nitrogen monoxide"[Title/Abstract] OR (“Monoxide"[Title/Abstract] AND “Nitrogen"[Title/Abstract]) OR “mononitrogen monoxide" [Title/Abstract] OR (“Monoxide"[Title/Abstract] AND “Mononitrogen"[Title/Abstract]))
**Scopus**	( TITLE-ABS-KEY ( “polycystic ovary syndrome" ) ) OR ( ( TITLE-ABS-KEY ( “ovary syndrome" ) AND TITLE-ABS-KEY ( polycystic ) ) ) OR ( ( TITLE-ABS-KEY ( syndrome ) AND TITLE-ABS-KEY ( “polycystic ovary" ) ) ) OR ( TITLE-ABS-KEY ( “stein-leventhal syndrome" ) ) OR ( TITLE-ABS-KEY ( “stein-leventhal syndrome" ) ) OR ( TITLE-ABS-KEY ( “stein leventhal syndrome" ) ) OR ( ( TITLE-ABS-KEY ( syndrome ) AND TITLE-ABS-KEY ( “stein-leventhal" ) ) ) OR ( TITLE-ABS-KEY ( “sclerocystic ovarian degeneration" ) ) OR ( ( TITLE-ABS-KEY ( “ovarian degeneration" ) AND TITLE-ABS-KEY ( “sclerocystic" ) ) ) OR ( TITLE-ABS-KEY ( “polycystic ovarian syndrome" ) ) OR ( ( TITLE-ABS-KEY ( “ovarian syndrome" ) AND TITLE-ABS-KEY ( polycystic ) ) ) OR ( TITLE-ABS-KEY ( “polycystic ovary syndrome 1" ) ) OR ( TITLE-ABS-KEY ( “sclerocystic ovaries" ) ) OR ( ( TITLE-ABS-KEY ( “ovary" ) AND TITLE-ABS-KEY ( “sclerocystic" ) ) ) OR ( TITLE-ABS-KEY ( “sclerocystic ovary" ) ) OR ( TITLE-ABS-KEY ( “pcos" ) ) OR ( TITLE-ABS-KEY ( “pco" ) ) AND ( TITLE-ABS-KEY ( “nitric oxide" ) ) OR ( ( TITLE-ABS-KEY ( nitric ) AND TITLE-ABS-KEY ( oxide ) ) ) OR ( ( TITLE-ABS-KEY ( “nitric oxide" ) AND TITLE-ABS-KEY ( “endothelium-derived" ) ) ) OR ( TITLE-ABS-KEY ( “endothelium-derived nitric oxide" ) ) OR ( ( TITLE-ABS-KEY ( “nitric oxide" ) AND TITLE-ABS-KEY ( “endothelium derived" ) ) ) OR ( TITLE-ABS-KEY ( “endogenous nitrate vasodilator" ) ) OR ( ( TITLE-ABS-KEY ( “nitrate vasodilator" ) AND TITLE-ABS-KEY ( “endogenous" ) ) ) OR ( ( TITLE-ABS-KEY ( “vasodilator" ) AND TITLE-ABS-KEY ( “endogenous nitrate" ) ) ) OR ( TITLE-ABS-KEY ( “nitrogen monoxide" ) ) OR ( ( TITLE-ABS-KEY ( “monoxide" ) AND TITLE-ABS-KEY ( “nitrogen" ) ) ) OR ( TITLE-ABS-KEY ( “mononitrogen monoxide" ) ) OR ( ( TITLE-ABS-KEY ( “monoxide" ) AND TITLE-ABS-KEY ( “mononitrogen" ) ) )
**Cochrane Library**	( (“polycystic ovary syndrome"):ti,ab,kw OR (“ovary syndrome"):ti,ab,kw OR ( (“ovary syndrome"):ti,ab,kw AND (polycystic):ti,ab,kw) OR ((syndrome):ti,ab,kw AND (“polycystic ovary"):ti,ab,kw) OR (“stein-leventhal syndrome"):ti,ab,kw OR (“stein leventhal syndrome"):ti,ab,kw OR ((syndrome):ti,ab,kw AND (“stein-leventhal"):ti,ab,kw) OR (“sclerocystic ovarian degeneration"):ti,ab,kw OR ( (“ovarian degeneration"):ti,ab,kw AND (“sclerocystic"):ti,ab,kw) OR (“polycystic ovarian syndrome"):ti,ab,kw OR ( (“ovarian syndrome"):ti,ab,kw AND (polycystic):ti,ab,kw) OR (“polycystic ovary syndrome 1"):ti,ab,kw OR (“sclerocystic ovaries"):ti,ab,kw OR ( (“ovary"):ti,ab,kw AND (“sclerocystic"):ti,ab,kw) OR (“sclerocystic ovary"):ti,ab,kw OR (“pcos"):ti,ab,kw OR (“pco"):ti,ab,kw) AND ( (“nitric oxide"):ti,ab,kw OR ((nitric):ti,ab,kw AND (oxide):ti,ab,kw) OR ( (“nitric oxide"):ti,ab,kw AND (“endothelium-derived"):ti,ab,kw) OR (“endothelium-derived nitric oxide"):ti,ab,kw OR ( (“nitric oxide"):ti,ab,kw AND (“endothelium derived"):ti,ab,kw) OR (“endogenous nitrate vasodilator"):ti,ab,kw OR ( (“nitrate vasodilator"):ti,ab,kw AND (“endogenous"):ti,ab,kw) OR ( (“vasodilator"):ti,ab,kw AND (“endogenous nitrate"):ti,ab,kw) OR (“nitrogen monoxide"):ti,ab,kw OR ( (“monoxide"):ti,ab,kw AND (“nitrogen"):ti,ab,kw) OR (“mononitrogen monoxide"):ti,ab,kw OR ( (“monoxide"):ti,ab,kw AND (“mononitrogen"):ti,ab,kw))
**Web of Science**	(TS= (“Polycystic Ovary Syndrome") OR TS= (“Ovary Syndrome") OR (TS= (“Ovary Syndrome") AND TS=(Polycystic)) OR (TS=(Syndrome) AND TS= (“Polycystic Ovary")) OR TS= (“Stein-Leventhal Syndrome") OR TS= (“Stein Leventhal Syndrome") OR (TS=(Syndrome) AND TS= (“Stein-Leventhal")) OR TS= (“Sclerocystic Ovarian Degeneration") OR (TS= (“Ovarian Degeneration") AND TS=(Sclerocystic)) OR TS= (“Polycystic Ovarian Syndrome") OR (TS= (“Ovarian Syndrome") AND TS=(Polycystic)) OR TS= (“Polycystic Ovary Syndrome 1") OR TS= (“Sclerocystic Ovaries") OR (TS=(Ovary) AND TS=(Sclerocystic)) OR TS= (“Sclerocystic Ovary") OR TS=(pcos) OR TS=(pco)) AND (TS= (“nitric oxide") OR (TS=(nitric) AND TS=(oxide)) OR (TS=(“nitric oxide”) AND TS= (“endothelium-derived")) OR TS= (“endothelium-derived nitric oxide") OR (TS= (“nitric oxide") AND TS= (“endothelium derived")) OR TS= (“endogenous nitrate vasodilator") OR (TS= (“nitrate vasodilator") AND TS= (“endogenous")) OR (TS=(vasodilator) AND TS= (“endogenous nitrate")) OR TS= (“nitrogen monoxide") OR (TS=(monoxide) AND TS=(nitrogen)) OR TS= (“mononitrogen monoxide") OR (TS=(monoxide) AND TS=(mononitrogen)))
**Embase**	('polycystic ovary syndrome':ti,ab,kw OR 'ovary syndrome':ti,ab,kw OR ('ovary syndrome':ti,ab,kw AND polycystic:ti,ab,kw) OR (syndrome:ti,ab,kw AND 'polycystic ovary':ti,ab,kw) OR 'stein-leventhal syndrome':ti,ab,kw OR 'stein leventhal syndrome':ti,ab,kw OR (syndrome:ti,ab,kw AND 'stein-leventhal':ti,ab,kw) OR 'sclerocystic ovarian degeneration':ti,ab,kw OR ('ovarian degeneration':ti,ab,kw AND 'sclerocystic':ti,ab,kw) OR 'polycystic ovarian syndrome':ti,ab,kw OR ('ovarian syndrome':ti,ab,kw AND polycystic:ti,ab,kw) OR 'polycystic ovary syndrome 1':ti,ab,kw OR 'sclerocystic ovaries':ti,ab,kw OR (ovary:ti,ab,kw AND 'sclerocystic':ti,ab,kw) OR 'sclerocystic ovary':ti,ab,kw OR 'pcos':ti,ab,kw OR 'pco':ti,ab,kw) AND ('nitric oxide':ti,ab,kw OR (nitric:ti,ab,kw AND oxide:ti,ab,kw) OR ('nitric oxide':ti,ab,kw AND 'endothelium-derived':ti,ab,kw) OR 'endothelium-derived nitric oxide':ti,ab,kw OR ('nitric oxide':ti,ab,kw AND 'endothelium derived':ti,ab,kw) OR 'endogenous nitrate vasodilator':ti,ab,kw OR ('nitrate vasodilator':ti,ab,kw AND 'endogenous':ti,ab,kw) OR (vasodilator:ti,ab,kw AND 'endogenous nitrate':ti,ab,kw) OR 'nitrogen monoxide':ti,ab,kw OR ('monoxide':ti,ab,kw AND 'nitrogen':ti,ab,kw) OR 'mononitrogen monoxide':ti,ab,kw OR ('monoxide':ti,ab,kw AND 'mononitrogen':ti,ab,kw))

### Study selection

After deleting the duplicate articles, 2 independent reviewers (S. B. and M. A.) assessed the eligibility based on inclusion/exclusion criteria and reviewed the titles/abstracts of the retrieved studies. Subsequently, 2 reviewers assessed the full texts, and disagreements were resolved with arbitration by the third reviewer.

### Data extraction

Data extraction was systematically carried out using a predefined checklist designed to capture essential elements relevant to the research question adhering to the study extraction design outlined in our previous research (18–21). The checklist included information on the study's origin, such as the first author's name, publication year, and geographic region of the study. Additionally, details on study design (cross-sectional, cohort, or case-control), participant characteristics (age, sample size, and body mass index), and measurements performed, diagnostic criteria, and reported outcomes were systematically extracted.

### Risk of bias assessment in the included studies

The Newcastle-Ottawa scale is a valuable tool for evaluating the quality of nonrandomized studies in a meta-analysis. Utilizing this scale ensures that only the most reliable studies are considered, leading to a more precise and trustworthy research analysis. The NOS is meticulously designed with 3 critical domains: the comparability of study groups, the selection of these groups, and the ascertainment of the outcome of interest. Each domain is equipped with specific criteria that guide the evaluation process. The scale ranges from 0–9, with studies scoring ≤ 4 classified as low quality and those scoring ≥ 5 as high quality. 2 independent reviewers reviewed each study and assessed it against the 3 domains. Any discrepancies were resolved with the assistance of a third reviewer.

### Statistical Analysis

The meta-analysis was conducted using Comprehensive Meta-Analysis v3.7z software. Circulating NO levels in women with PCOS were compared to those in control groups using the standardized mean difference (SMD) and a 95% confidence interval (CI). The utilization of SMD proved to be pivotal as it facilitated consistent comparison of effect sizes across studies employing diverse measurement scales for NO levels. Heterogeneity among studies was assessed using Cochran's Q test and the I^2^ statistic, with statistical significance set at p < 0.05 or I^2^ > 50%. A random-effects model was employed for data synthesis, considering potential variability among studies. Publication bias was assessed using Egger's tests, with significance set at a p-value of 0.05. Subgroup analyses and meta-regression analyses were conducted to explore potential sources of heterogeneity, such as age, geographical region, and study design. Furthermore, a sensitivity analysis was performed to evaluate the impact of individual studies by systematically excluding each study from the analysis. The overall statistical significance level was set at p < 0.05 for all the analyses as well as their prediction intervals were reported.

## 3. Results

### Characteristics of the included studies

**Figure 2:**
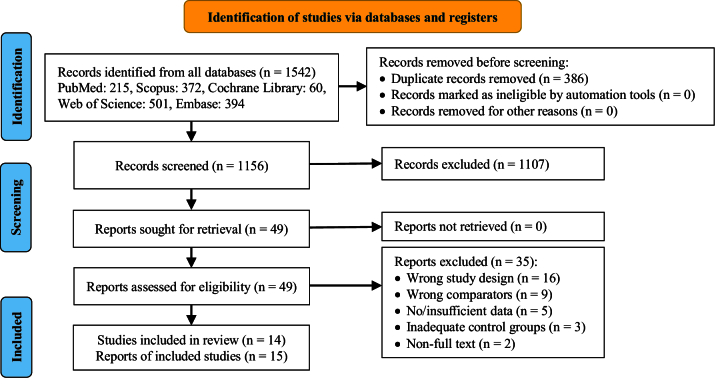
Flow diagram of study selection adjusted by PRISMA.

The systematic search strategy identified 1542 articles in the database. After removing 386 duplicate records and excluding 1107 articles based on title and abstract evaluation, 49 articles underwent a comprehensive full-text assessment. During this process, 35 studies were excluded for various reasons, including lack of relevant data on NO levels, insufficient sample size, failure to meet the diagnostic criteria for PCOS, and methodological flaws such as poor study design or inadequate control groups. Ultimately, 14 studies met the pre-established selection criteria involving a combined total of 1171 participants (Figure 2). The characteristics of these 14 studies, including participant demographics, sample type, and study design, are summarized in table II. Furthermore, the quality assessment of the included studies is presented in table III.

**Table 2:** Characteristics of the studies included in the systematic review and meta-analysis

**Author, yr (Ref)**	**Country**	**Ethnicity**	**Study designs**	**Number of participants**	**POCS diagnostic criteria**	**Age (yr) (PCOS vs. controls)**	**BMI (kg/m^2^) (PCOS vs. controls)**	**Sample (unit)**
**Lakshmi ** ***et al.*** **, 2023 (22)**	India	Asian	Case control	PCOS: 150Control: 100	Rotterdam	24.87 ± 4.73 24.38 ± 5.27	27.02 ± 6.03 24.31 ± 4.93	Serum ( μ mol/l)
**Yesil ** ***et al.*** **, 2022 (23)**	Turkey	Mixed	Cross sectional	PCOS: 45Control: 42	Rotterdam	22.11 ± 6.16 23.76 ± 5.34	25.37 ± 6.21 22.10 ± 3.44	Serum ( μ mol/l)
**Kohzadi ** ***et al.*** **, 2020 (24)**	Iran	Asian	Cross sectional	PCOS: 40 Control: 40	Rotterdam	30.70 ± 6.14 32.02 ± 6.24	26.66 ± 4.24 25.33 ± 3.15	Serum ( μ mol/l)
**Krishna ** ***et al.*** **, 2017 (25)**	Turkey	Mixed	Case control	PCOS: 29 Control: 20	Rotterdam	28 ± 2.52 28.6 ± 2.01	24.96 ± 3.22 23.44 ± 2.03	Plasma ( μ mol/l)
**Kocer ** ***et al.*** **, 2014 (26)**	Turkey	Mixed	Case control	PCOS: 15 Control: 17	NIH	22.73 ± 5.93 25.70 ± 5.14	23.76 ± 4.44 24.44 ± 1.28	Serum ( μ mol/l)
**Yavuz Taşlipinar** ***et al.*** **, 2014 (27)**	Turkey	Mixed	Case control	PCOS: 25 Control: 25	Rotterdam	24.64 ± 4.93 26.32 ± 3.89	26.35 ± 7.05 22.46 ± 3.43	Serum ( μ mol/l)
**Willis ** ***et al.*** **, 2014 (28)**	UK	European	Case control	PCOS: 17 Control: 18	Rotterdam	30 ± 6 29 ± 6	31 ± 6 31 ± 7	Plasma ( μ mol/l)
**Su ** ***et al.*** **, 2012 (29)**	China	Asian	Case control	PCOS: 13 Control: 12	NIH	N/A	27.80 ± 5.87 26.70 ± 4.05	Plasma ( μ mol/l)
**Karadeniz ** ***et al.*** **, 2011 (30)**	Turkey	Mixed	Case control	PCOS: 98 Control: 93	Rotterdam	25.63 ± 7.67 24.44 ± 5.69	24.25 ± 7.68 24.80 ± 5.88	Serum ( μ mol/l)
**Bayram ** ***et al.*** **, 2012 (31)**	Turkey	Mixed	Case control	PCOS: 45 Control: 17	Rotterdam	23.8 ± 4.2 21.4 ± 1.2	23.8 ± 4.2 21.4 ± 1.2	Serum ( μ mol/l)
**Baskol ** ***et al.*** **, 2012 (32)**	Turkey	Mixed	Case control	PCOS: 30 Control: 20	Rotterdam	28.2 ± 4.5 29.5 ± 4.5	26.1 ± 3.1 25.1 ± 4.8	Serum ( μ mol/l)
**Türkçüoğlu** ***et al.*** **, 2011 (33)**	Turkey	Mixed	Case control	PCOS: 18 Control: 12	Rotterdam	29.6 ± 6.2 30 ± 8	28.3 ± 2.4 29.4 ± 4.2	Serum ( μ mol/l)
**Erdogan ** ***et al.*** **, 2008 (34)**	Turkey	European	Case control	PCOS: 17 Control: 18	Rotterdam	24.02 ± 1.30 25.02 ± 1.25	23.33 ± 4.11 23.37 ± 4.10	Serum ( μ mol/l)
**Guan ** ***et al.*** **, 2008 (35)**	China	Asian	Case control	PCOS: 15 Control: 15	NIH	25.14 ± 3.63 27.41 ± 1.79	N/A	Plasma ( μ mol/l)
BMI: Body mass index, PCOS: Polycystic ovary syndrome, Ref: Reference, NIH: National Institutes of Health Criteria, N/A: Not available. Please note that some studies have examined more than one biomarker								

**Table 3:** Quality assessment based on the Newcastle-Ottawa Scale of studies included in this meta-analysis

**Author, yr (Ref)**	**Selection**				**Comparability**	**Exposure**			**Score**
	**An adequate definition of case**	**Representativeness of the case**	**Selection of controls**	**Definition of controls**	**Cases and controls matched and/or adjusted by factors**	**Assessment of exposure**	**The same method of ascertainment for cases and controls**	**The same response rate for both groups**	
**Lakshmi ** ***et al.*** **, 2023 (22)**	★	★	★	★	★	★	★	★	8
**Yeşil** ***et al.*** **, 2022 (23)**	★	★	★	–	★	★	★	★	7
**Kohzadi ** ***et al.*** **, 2020 (24)**	★	★	★	–	★	★	★	★	7
**Krishna ** ***et al.*** **, 2017 (25)**	★	★	★	★	★	★	★	★	8
**Kocer ** ***et al.*** **, 2014 (26)**	★	★	★	★	★★	–	★	–	7
**Yavuz Taşlipinar ** ***et al.*** **, 2014 (27)**	★	★	–	★	★	★	★	–	6
**Willis ** ***et al.*** **, 2014 (28)**	★	★	–	★	★	★	★	★	7
**Su ** ***et al.*** **, 2012 (29)**	–	★	★	★	★	–	★	–	5
**Karadeniz ** ***et al.*** **, 2011 (30)**	–	★	–	★	★	★	★	-	5
**Bayram ** ***et al.*** **, 2012 (31)**	★	★	★	★	★	★	★	★	8
**Baskol ** ***et al.*** **, 2012 (32)**	★	★	★	–	★	★	★	–	6
**Türkçüoğlu ** ***et al.*** **, 2011 (33)**	★	★	★	★	★★	–	★	–	7
**Erdogan ** ***et al.*** **, 2008 (34)**	–	★	★	–	★	–	★	★	5
**Guan ** ***et al.*** **, 2008 (35)**	★	★	–	★	★★	★	★	–	7
★ : A study can be awarded a maximum of one star for each numbered item, except for the item `control for important factor or additional factor.' ★★ : A maximum of 2 stars can be awarded for `control for important factor or additional factor'									

### Association and comparison details

A comprehensive investigation, encompassing 14 studies (15 reports) with a total of 1171 participants, was conducted to examine the correlation between NO levels and PCOS. Subgroup and meta-regression analyses were performed to explore potential sources of heterogeneity, including BMI, age, type of sample, diagnostic criteria for PCOS, and study year. This thorough analysis significantly enhanced our understanding of the association between NO levels and PCOS, illuminating the factors contributing to the observed heterogeneity.

### Relationship between serum NO levels and PCOS 

#### Meta-analysis 

**Figure 3:**
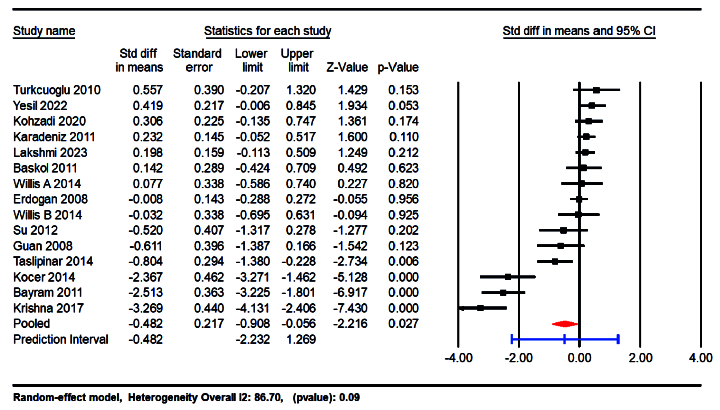
The forest plots compare circulating NO levels between PCOS and control groups.

The meta-analysis results indicated a significant reduction in NO levels in the group with PCOS compared to the control group, with an SMD of -0.482 (95% CI: -0.908 to -0.056; p = 0.027). This finding suggests a substantial decrease in NO levels associated with PCOS. Furthermore, a high degree of heterogeneity was detected among the included studies, as evidenced by an I^2^ value of 91.34% and a p-value of 0.01, indicating considerable variability in the study results (Figure 3).

#### Prediction interval 

**Figure 4:**
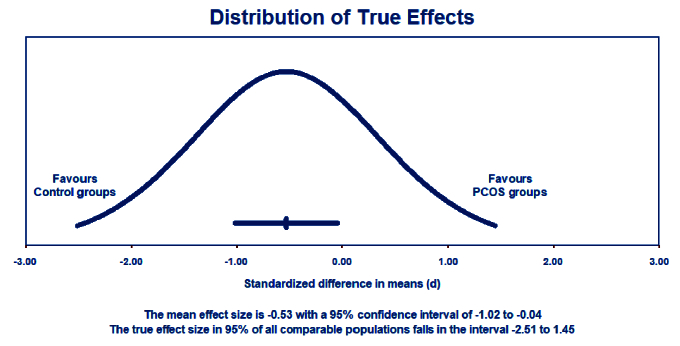
The prediction interval SMD of NO levels between PCOS and control groups.

The estimation of a prediction interval revealed a range of -2.51 to 1.45 for the true effect size, assuming a normal distribution. This interval is expected to encompass the effect size in 95% of comparable populations. However, it is important to note that variations in the actual effect size may occur within this interval due to factors such as sample size and study design. Therefore, careful consideration is warranted when interpreting results within this prediction interval (Figure 4).

#### Subgroup analysis

**Figure 5:**
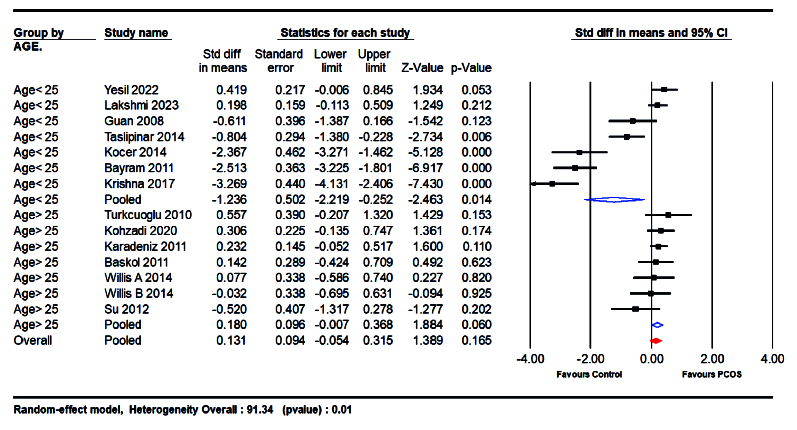
Forest plot of age > 25 yr and ≤ 25 yr in subgroup analysis.

Our systematic review and meta-analysis revealed a significant association between NO levels and PCOS, with nuanced variations observed across different subgroups. The subgroup analyses focused on age, BMI, sample type, and race, providing valuable insights into the diverse manifestations of NO levels in PCOS patients. A noteworthy difference was observed between women aged 25 yr and above and those < 25 yr. The studies were analyzed in 2 subgroups, one including participants with an average age of ≥ 25 yr (SMD = 0.180; 95% CI: -0.007 to 0.368; p = 0.060), and the other including those < 25 yr (SMD = -1.236; 95% CI: -2.219 to -0.252; p = 0.014). According to the analysis, individuals aged ≥ 25 had higher levels of NO (Figure 5).

**Figure 6:**
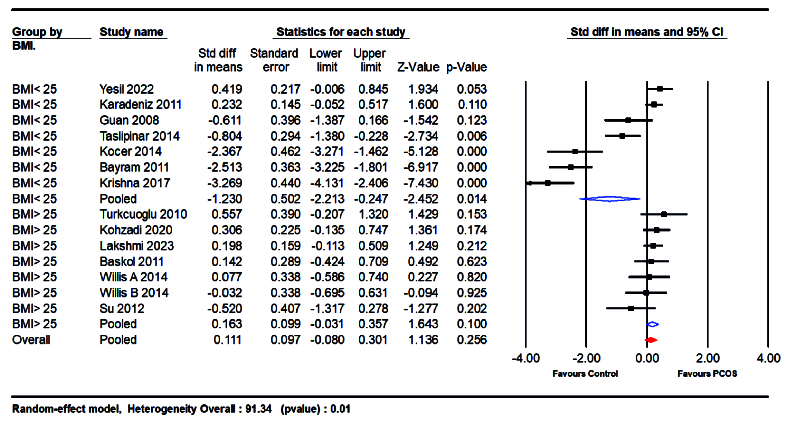
Forest plot for subgroup analysis comparing BMI categories: BMI > 25 kg/m^2^ and BMI ≤ 25 kg/m^2^.

Moreover, subgroup analyses based on BMI and the sample type were conducted. The analysis of studies with a mean BMI of ≥ 25 kg/m^2^ or < 25 kg/m^2^ revealed that women with BMI > 25 kg/m^2^ had higher NO levels (SMD = 0.163; 95% CI: -0.031 to 0.357; p = 0.10) compared to those with BMI < 25 kg/m^2^ (SMD = -1.230; 95% CI: -2.213 to -0.247; p = 0.014) (Figure 6).

**Figure 7:**
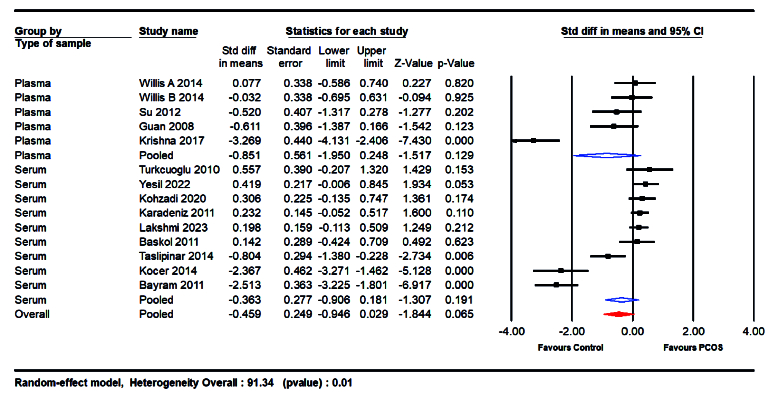
Forest plot of sample type based on serum or plasma in subgroup analysis.

In addition, the study also looked at subgroups based on the type of sample collected (serum or plasma). The results showed that women with plasma samples had lower levels of NO (SMD = -0.851; 95% CI: -1.950 to 0.248; p = 0.129) compared to those with serum samples (SMD = -0.363; 95% CI: -0.906 to 0.181; p = 0.19) (Table IV, Figure 7).

**Table 4:** Subgroup meta-analysis of the included studies

**Subgroup analysis**		**Number of studies**	**SMD (95% CI)**	**I^2^**	**P-value for heterogeneity**
**BMI**					
	**< 25**	7	-0.387 (-0.58, -0.19)	95.38	< 001
	**≥ 25**	7	0.16 (-0.03, 0.35)	0.00	0.59
**Mean age**					
	**< 25**	7	-0.450 (-0.65, -0.25)	95.19	< 001
	**≥ 25**	7	0.180 (-0.00, 0.36)	0.00	0.52
**Sample type**					
	**Plasma**	5	-0.671 (-1.00, -0.34)	90.83	< 001
	**Serum**	9	-0.001 (-0.15, 0.14)	91.45	< 001
**Ethnicity**					
	**Asian **	4	-0.045 (-0.27, 0.18)	95.16	< 001
	**European**	2	0.191 (-0.07, 0.45)	0.00	0.473
	**Mixed**	8	-0.404 (-0.62, -0.18)	90.73	< 001
A random-effects model was employed for data synthesis. BMI: Body mass index, SMD: Standardized mean difference, CI: Confidence interval					

**Figure 8:**
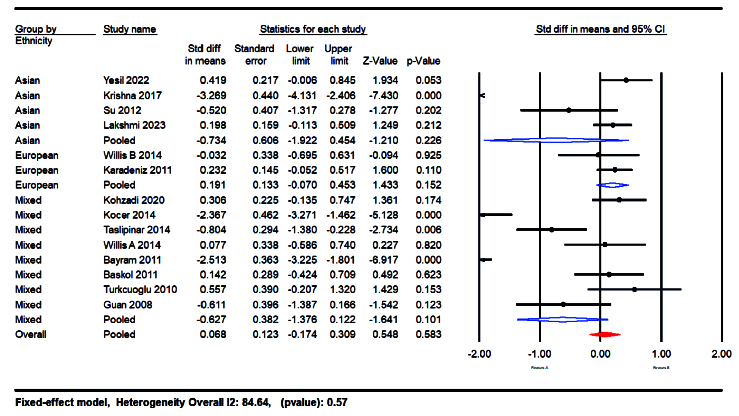
Forest plot that presents the results of the subgroup analysis based on ethnicity.

Subgroup analyses were conducted based on ethnicity, including Asian, European, and mixed races. When focusing on racial diversity, studies revealed that women of European ethnicity had higher NO levels (SMD = 0.191; 95% CI: -0.07 to 0.45; p = 0.015) compared to those of Asian (SMD = -0.734; 95% CI: -1.92 to -045; p = 0.22) and mixed race (SMD = -0.62; 95% CI: -1.641 to 0.101; p = 0.10) (Figure 8).

#### Meta-regression analysis

A meta-regression analysis was conducted to investigate the relationship between sample size, PCOS diagnostic criteria, study year, BMI, and overall effect size. The analysis results indicated no association between PCOS diagnostic criteria (meta-regression coefficient: 0.698; 95% CI: -0.619 to 2.016; p = 0.29), sample size (meta-regression coefficient: 0.064; 95% CI: -0.006 to 0.198; p = 0.11), and year of studies (meta-regression coefficient: 0.015; 95% CI: -0.138 to 0.168; p = 0.84) with effect size. However, the analysis revealed a significant positive relationship between BMI and effect sizes (meta-regression coefficient: 0.327; 95% CI: 0.051 to 0.604; p = 0.02).

These findings suggest that BMI can significantly impact on the effect size. In contrast, factors such as PCOS diagnostic criteria, sample size, and year of study did not significantly affect the results of the meta-analysis studies.

### Sensitivity analysis and publication bias

Sensitivity analysis, excluding studies one by one, demonstrated a stable SMD range from -0.696 to -0.383. The 95% CI ranged from -1.326 to 0.095. The values of I^2^ maintained clarity, indicating stable results throughout, with no significant changes observed. The funnel plot method, utilized for detecting publication bias, and Egger's test, used for quantifying publication bias, did not provide evidence of publication bias regarding the included studies (coefficient: -6.061; 1.963; 95% CI: -10.901 to 1.022, p = 0.112). These results further support the credibility and robustness of the pooled findings.

## 4. Discussion

### Meta-analysis overview

This comprehensive meta-analysis examines the association between circulating NO levels and PCOS, synthesizing data from a diverse array of studies. The analysis encompasses a significant sample size, integrating findings from 1171 participants across 14 studies. The results consistently reveal a notable decrease in NO levels among individuals with PCOS compared to healthy controls. This finding is pivotal, suggesting that NO may serve as a potential biomarker for diagnosing and managing PCOS. This discussion delves into the biological mechanisms underlying these observations, interpreting the main findings, and exploring their implications for clinical practice and future research.

### Interpretation of main findings

Our meta-analysis highlights a significant reduction in circulating NO levels in women with PCOS, with an SMD of -0.482 (95% CI: -0.908 to -0.056; p = 0.027). This suggests a strong association between low NO levels and the presence of PCOS. The substantial heterogeneity observed (I^2^ = 91.34%) indicates variability in NO levels based on demographic and clinical factors, warranting further investigation. Moreover, the prediction interval (-2.51 to 1.45) underscores the variability in effect size, suggesting that while reduced NO levels are generally associated with PCOS, the specific utility of NO as a biomarker may vary across different populations and clinical settings.

### Subgroup analysis reflection: Interpreting variability and trends

#### Age-based analysis

The differential impact of NO levels across various age groups elucidates critical aspects of the pathogenesis of PCOS. Among women over 30 yr old, NO levels were significantly elevated in those with PCOS compared to the control group (SMD = 0.180 vs. -1.236). This increase may reflect age-related changes in vascular function or the prolonged exposure to metabolic and endocrine disturbances typical of PCOS. In contrast, younger women (aged 16–25) exhibited a less pronounced reduction in NO levels. This difference may indicate an alternative pathogenic mechanism or an adaptive response specific to younger individuals.

#### BMI-based analysis

BMI significantly moderated the relationship between NO levels and PCOS. Women with a BMI greater than 30 kg/m^2^ exhibited a more substantial decrease in NO levels compared to those with a BMI below 25 kg/m^2^ (SMD = 0.163 vs. -1.230). This suggests that obesity, a common feature of PCOS, may exacerbate endothelial dysfunction and reduce NO bioavailability. The association between obesity and NO levels underscores the importance of considering BMI in the clinical assessment and management of PCOS.

#### Sample type and ethnicity-based analysis

The analysis did not find significant differences in NO levels when comparing serum and plasma samples (SMD = -0.851 vs. -0.906). This suggests that the bioavailability of NO and its potential as a biomarker is consistent across different sample types, adding robustness to the findings and allowing flexibility in clinical assessments. Subgroup analyses based on ethnicity revealed that women of European ethnicity had higher NO levels (SMD = 0.191; p = 0.473) compared to those of Asian (SMD = -0.045; p ≤ 001) and mixed race (SMD = -0.404 p ≤ 001). These findings suggest that genetic and environmental factors related to ethnicity may influence NO levels and their association with PCOS.

### Deciphering study outcome patterns: Meta-regression insights

The meta-regression analysis provided vital insights into the factors influencing circulating NO levels in patients with PCOS. The analysis examined variables such as PCOS diagnostic criteria, sample size, study year, and BMI, with significant findings, particularly regarding BMI.

The meta-regression revealed no statistically significant association between the effect size and PCOS diagnostic criteria, sample size, and study year. These results suggest that these factors do not significantly impact the heterogeneity of NO levels reported across studies, indicating a degree of consistency in findings despite variations in study designs, populations, and periods. However, a significant positive relationship was observed between BMI and effect size (meta-regression coefficient: 0.327; 95% CI: 0.051 to 0.604; p = 0.02). This finding implies that BMI is a crucial determinant of circulating NO levels in PCOS patients. Higher BMI appears to be associated with increased NO levels, which could reflect the interplay between obesity, metabolic dysfunction, and OS commonly seen in PCOS.

The role of BMI in modulating NO levels can be understood through several mechanisms. Obesity is often associated with insulin resistance, a hallmark of PCOS, which is linked to endothelial dysfunction and altered NO production. Adipose tissue in obese individuals secretes pro-inflammatory cytokines and free fatty acids, which can further impair endothelial function and exacerbate OS. This cascade of metabolic disturbances can lead to increased NO production as a compensatory mechanism or as a marker of ongoing OS.

### Prior research and contextualization

PCOS is recognized as a multifactorial endocrine disorder, which represents a primary cause of infertility issues among women of reproductive age (36, 37). Considering several factors influencing NO levels, this review provides a critical analysis of the role of NO as a potential biomarker in the pathology of PCOS. The intricate association between NO levels and PCOS, as suggested by the results of this meta-analysis, underscores the complexity of the disorder. NO is a well-known endothelium-derived relaxing factor that plays a crucial role in vascular homeostasis (27).

NO is a well-established endothelium-derived relaxing factor essential for maintaining vascular homeostasis. It mediates vasodilation by stimulating the soluble guanylate cyclase in vascular smooth muscle cells, leading to cyclic guanosine monophosphate (cGMP) production and subsequent relaxation of smooth muscle fibers. This vasodilatory effect is crucial for regulating blood flow and blood pressure (38). Reduced NO bioavailability is associated with endothelial dysfunction, a precursor to atherosclerosis and cardiovascular disease (CVD) (39).

In the context of PCOS, several studies have identified a link between endothelial dysfunction and the syndrome's pathophysiology. For example, a study by Paradisi et al. found that women with PCOS exhibited impaired endothelial function, as evidenced by decreased flow-mediated dilation of the brachial artery (40). This dysfunction is thought to stem from reduced NO availability, contributing to the increased cardiovascular risk observed in PCOS patients.

Insulin resistance is a core feature of PCOS, affecting approximately 50–70% of women with the condition (41, 42). NO plays a significant role in insulin signaling and glucose metabolism. Insulin stimulates NO production via the PI3K/Akt pathway, which promotes glucose uptake in peripheral tissues (43). However, hyperinsulinemia, commonly seen in PCOS, can impair eNOS activity, reducing NO synthesis and exacerbating insulin resistance (44).

Clinical studies have demonstrated a correlation between reduced NO levels and insulin resistance in PCOS. For instance, a study by Yildirim reported significantly lower NO levels in insulin-resistant women with PCOS compared to healthy controls (45). This reduction was associated with elevated fasting insulin and glucose levels, underscoring the role of NO in metabolic regulation and its potential as a biomarker for insulin resistance in PCOS.

Chronic low-grade inflammation is a hallmark of PCOS, characterized by elevated levels of inflammatory cytokines such as tumor necrosis factor-alpha and interleukin-6 (46). These cytokines can inhibit eNOS expression and activity, leading to reduced NO production. Additionally, OS, indicated by increased ROS levels, further diminishes NO bioavailability by converting NO to peroxynitrite, a reactive nitrogen species (47). Previous research has provided mixed results regarding NO levels in PCOS, with some studies reporting decreased levels while others found no significant differences. A study by Macut et al. for example, reported significantly lower NO levels in women with PCOS, which was associated with increased insulin resistance and cardiovascular risk (48).

### Elucidating the biological mechanisms of NO in the pathophysiology of PCOS

The underlying mechanisms connecting NO levels to PCOS involve both the factors that lead to reduced NO levels and the subsequent impacts on the disease's progression. In PCOS, reduced NO bioavailability is a marker of endothelial dysfunction, which is a precursor to atherosclerosis and other CVDs (39). Several inter-related mechanisms drive this endothelial dysfunction in PCOS. Hyperinsulinemia, a common feature in PCOS, leads to reduced NO production by inhibiting eNOS activity. Elevated insulin levels in insulin-resistant PCOS patients negatively affect eNOS via the phosphoinositide 3-kinase (PI3K) pathway, thus decreasing NO synthesis (43). Insulin resistance, prevalent in PCOS, impairs the PI3K/Akt pathway, which is critical for eNOS activation and NO production. This impairment leads to decreased NO availability and endothelial dysfunction (49). Hyperandrogenism, another hallmark of PCOS, further contributes to endothelial dysfunction by downregulating eNOS expression and impairing NO-mediated vasodilation (50). Elevated androgen levels in PCOS increase the production of asymmetric dimethylarginine, an endogenous inhibitor of eNOS, thus reducing NO synthesis (51).

The relationship between NO and insulin resistance is complex and bidirectional. NO facilitates insulin-mediated glucose uptake in skeletal muscle by enhancing blood flow and glucose delivery to peripheral tissues (52). NO enhances the translocation of glucose transporter type 4 to the cell membrane in response to insulin, promoting glucose uptake (53). In PCOS, decreased NO availability due to endothelial dysfunction and OS impairs this process, exacerbating insulin resistance. This insulin resistance perpetuates hyperinsulinemia, creating a vicious cycle of metabolic dysregulation (54). Furthermore, insulin resistance in PCOS is linked to adipose tissue dysfunction.

Adipocytes in PCOS patients exhibit impaired NO synthesis, leading to reduced adiponectin secretion and increased secretion of pro-inflammatory cytokines (55). This adipose tissue inflammation reduces insulin sensitivity and increases systemic OS, subsequently reducing NO levels and exacerbating metabolic outcomes in PCOS. Elevated free fatty acids in obesity, often seen in PCOS, also play a role. Free fatty acids induce mitochondrial dysfunction and increase ROS production in skeletal muscle, which inhibits the insulin signaling pathway and reduces NO production, further contributing to insulin resistance (56, 57).

NO plays a pivotal role in ovarian physiology, particularly in follicular development and ovulation. NO regulates ovarian blood flow, follicle maturation, and oocyte quality (58). In PCOS, reduced NO levels can impair these processes, leading to anovulation and the formation of ovarian cysts, hallmark features of the syndrome. Furthermore, the presence of hyperinsulinemia and hyperandrogenism in individuals with PCOS disturbs the microenvironment of the ovaries, leading to a decrease in the synthesis of NO and hindering the process of follicular angiogenesis.

This leads to poor follicular development and increased follicular atresia (58). Insulin resistance also reduces the bioavailability of NO in the ovaries, further impairing folliculogenesis (59). The ovarian dysfunction in PCOS is also linked to the dysregulation of steroidogenesis. NO modulates the production of steroid hormones by influencing the expression of key enzymes involved in steroid biosynthesis. Reduced NO levels lead to an imbalance in androgen and estrogen production, contributing to the hyperandrogenic state seen in PCOS (60).

Additionally, the dysregulation of the NO-cGMP pathway in the ovaries of PCOS patients contributes to impaired ovulation. NO activates guanylate cyclase, increasing cGMP levels crucial for follicular expansion and oocyte maturation. Reduced NO levels disrupt this signaling pathway, leading to ovulatory dysfunction (61).

### Clinical implications and future directions

The significant association between reduced NO levels and PCOS suggests that NO could serve as a valuable biomarker for the early diagnosis and management of PCOS. Monitoring NO levels could help identify individuals at risk of developing PCOS-related complications, such as CVD and insulin resistance.

Understanding the mechanistic role of NO in PCOS could open new avenues for therapeutic strategies aimed at modulating NO levels to mitigate disease severity. For instance, interventions that enhance NO production, such as lifestyle modifications (e.g., exercise and diet) or pharmacological agents (e.g., L-arginine supplementation), could improve endothelial function and insulin sensitivity in PCOS patients.

Future research should focus on longitudinal studies to track NO levels over time in women with PCOS, from initial diagnosis through treatment and disease progression. Such studies could clarify the temporal dynamics of NO expression and its prognostic utility. Additionally, interventional studies examining the effects of therapies targeting NO pathways could provide insights into their potential benefits in managing PCOS.

### Strengths and limitations

This study boasts several strengths, including a rigorous search methodology, a substantial sample size, and consistent findings across multiple studies. The use of subgroup analyses and meta-regression techniques has enhanced the interpretation of results, adding depth to our understanding of the relationship between NO levels and PCOS. However, several limitations warrant acknowledgment. The variability in detection methods across included studies may introduce discrepancies in reported NO levels. Despite efforts to standardize our analysis, differences in assay sensitivity and specificity could influence the consistency of results. Additionally, the predominance of English-language articles in the search could mask relevant data in non-English publications, potentially introducing language bias.

The heterogeneity in study designs and data collection methods across studies adds another layer of complexity to data interpretation. Variations in participant lifestyles, such as diet, physical activity, and smoking status, could influence NO levels and were not uniformly accounted for in all studies. Furthermore, the evolving landscape of PCOS diagnostic criteria and treatment modalities poses additional challenges in standardizing findings.

## 5. Conclusion 

This meta-analysis reviewed data from 14 studies and 1171 participants, highlighting the potential of NO levels as a biomarker for PCOS and suggesting an association between NO dysregulation and PCOS pathophysiology. Our findings underscore the pertinence of NO levels as a potential biomarker for PCOS, suggesting an interplay between NO dysregulation and the pathophysiology of PCOS. Significantly, our research adds a critical dimension to the clinical understanding of PCOS, aligning with the hypothesis that NO could serve not only as a marker of disease presence but also as an indicator of its severity. This relationship holds promising outcomes for developing more precise diagnostic criteria and improving the therapeutic strategies tailored to individual NO profiles. It also proposes a new vista for clinicians to prognosticate the progression and mitigate the risks associated with PCOS by monitoring NO levels. Future research should aim at longitudinal studies with larger cohorts and standardized methodologies for assessing NO levels, to advance beyond correlation toward a clearer understanding of causation.

##  Data Availability

Data supporting the findings of this study are available upon reasonable request from the corresponding author.

##  Author Contributions

SS. Bahreiny and A. Ahangarpour contributed to data analysis, manuscript preparation, and supervision, while R. Fard, M. Aghaei, and A. Ahangarpour contributed to manuscript searching, preparation, and data analysis. E. Harooni and M. Amraei contributed to the search strategy, article searching, and manuscript preparation. The manuscript has been reviewed and approved by all authors.

##  Conflict of Interest

The authors declare that there is no conflict of interest.
